# Quercetin Suppresses Cyclooxygenase-2 Expression and Angiogenesis through Inactivation of P300 Signaling

**DOI:** 10.1371/journal.pone.0022934

**Published:** 2011-08-08

**Authors:** Xiangsheng Xiao, Dingbo Shi, Liqun Liu, Jingshu Wang, Xiaoming Xie, Tiebang Kang, Wuguo Deng

**Affiliations:** 1 State Key Laboratory of Oncology in South China, Sun Yat-Sen University Cancer Center, Guangzhou, China; 2 The First Affiliated Hospital-Huangpu Hospital, Sun Yat-Sen University, Guangzhou, China; The Chinese University of Hong Kong, Hong Kong

## Abstract

Quercetin, a polyphenolic bioflavonoid, possesses multiple pharmacological actions including anti-inflammatory and antitumor properties. However, the precise action mechanisms of quercetin remain unclear. Here, we reported the regulatory actions of quercetin on cyclooxygenase-2 (COX-2), an important mediator in inflammation and tumor promotion, and revealed the underlying mechanisms. Quercetin significantly suppressed COX-2 mRNA and protein expression and prostaglandin (PG) E(2) production, as well as COX-2 promoter activation in breast cancer cells. Quercetin also significantly inhibited COX-2-mediated angiogenesis in human endothelial cells in a dose-dependent manner. The *in vitro* streptavidin-agarose pulldown assay and *in vivo* chromatin immunoprecipitation assay showed that quercetin considerably inhibited the binding of the transactivators CREB2, C-Jun, C/EBPβ and NF-κB and blocked the recruitment of the coactivator p300 to COX-2 promoter. Moreover, quercetin effectively inhibited p300 histone acetyltransferase (HAT) activity, thereby attenuating the p300-mediated acetylation of NF-κB. Treatment of cells with p300 HAT inhibitor roscovitine was as effective as quercetin at inhibiting p300 HAT activity. Addition of quercetin to roscovitine-treated cells did not change the roscovitine-induced inhibition of p300 HAT activity. Conversely, gene delivery of constitutively active p300 significantly reversed the quercetin-mediated inhibition of endogenous HAT activity. These results indicate that quercetin suppresses COX-2 expression by inhibiting the p300 signaling and blocking the binding of multiple transactivators to COX-2 promoter. Our findings therefore reveal a novel mechanism of action of quercetin and suggest a potential use for quercetin in the treatment of COX-2-mediated diseases such as breast cancers.

## Introduction

Quercetin is a dietary polyphenolic flavonoid found in many fruits, vegetables, nuts, and red wine, and exerts diverse biological activities including anti-inflammatory and antitumor properties [1]–[6]. It possesses chemotherapeutic potential in various cancers, and be capable of modulating several signal transduction pathways associated with cell survival, proliferation and apoptosis [7]–[20]. Previous study has shown that quercetin inhibits tumor necrosis factor-*α* (TNF)-induced NF-κB transcription factor recruitment to proinflammatory gene promoters in the murine small intestinal epithelial cells [6]. Quercetin inhibited TNF-induced interferon-δ-inducible protein 10 (IP-10) and macrophage inflammatory protein 2 (MIP-2) gene expression by inhibiting histone acetyltransferase (HAT) activity and histone 3 (H3) acetylation/phosphorylation as well as blocking phospho-Rel A (NF-κB p65) and cofactor CBP/p300 binding to the IP-10 and MIP-2 gene promoters. These studies support an anti-inflammatory effect of quercetin in epithelial cells through mechanisms that inhibit NF-κB and cofactor recruitment at the chromatin of proinflammatory genes.

Cyclooxygenase-2 (COX-2) is an inducible enzyme which plays a critical role in multiple pathophysiological processes including inflammation, atherosclerosis, tissue injury, angiogenesis and tumorigenesis [21]–[24]. COX-2 catalyzes the conversion of arachidonic acid to prostaglandin H_2_, which is further converted to biologically active prostaglandins and thromboxane A_2_ (TXA_2_) by specific enzymes [25]–[27]. Abnormal expression of cyclooxygenase-2 (COX-2) is an important mediator in inflammation and tumor promotion. It has been shown that overexpression of COX-2 is significantly correlated to invasiveness, prognosis, and survival in some cancers [28]–[30]. Inhibition of COX-2 with selective COX-2 inhibitors effectively prevents inflammation, proliferation and angiogenesis, and induces apoptosis in human cells. Importantly, COX-2 inhibitors have been shown to act additively or synergistically with currently used chemotherapeutic and targeted agents [31]–[33].

COX-2 transcriptional regulation has been extensively characterized. A core promoter region within 500 bp from the COX-2 transcription start site harbors several regulatory elements notably cyclic AMP response element (CRE), CCAAT/enhancer binding protein (C/EBP) enhancer element and NF-κB binding sites, that are essential for COX-2 promoter activity in response to inflammatory signals [34], [35] . Binding of multiple transactivators to their respective cis-acting elements on the core COX-2 promoter results in overexpression of COX-2. Importantly, p300 has been shown to exert a global effect on COX-2 promoter chromatin structure, which is able to enhance the binding of transactivators to COX-2 promoter. The mechanism by which COX-2 is highly expressed in tumorigenesis and angiogenesis is not completely understood.

COX-2 expression is reported to be abrogated by an array of small-molecule compounds such as melatonin and salicylate [36], [37]. Numerous studies have also demonstrated the inhibitory effects of quercetin on COX-2 expression [38]–[40]. Although quercetin has been shown to inhibit TNF-induced expression of the proinflammatory genes IP-10 and MIP-2 by targeting the NF-*κ*B signaling pathway and modulating H3 acetylation in murine small intestinal epithelial cells [6], the excise molecular mechanisms by which quercetin inhibits expression of the specific proinflammatory gene such as COX-2 in human cancer cells remained unclear.

In this study, we investigated the mechanisms of action of quercetin for COX-2 suppression in human breast cancer cells and demonstrated that quercetin specifically targeted at p300 signaling pathway to regulate COX-2 expression and COX-2-mediated angiogenesis. We found that quercetin suppressed p300 HAT activity, abolished the p300-mediated NF-κB p50 acetylation, and blocked binding of p300 and the multiple transactivators such as CREB2, C-Jun, C/EBPβ and NF-κB to COX-2 gene promoter in human breast cancer cells. Our study therefore reveals a novel molecular mechanism by which quercetin inhibits COX-2 expression in human cancer cells and suggests a potential use for quercetin in the treatment of COX-2-mediated diseases such as breast cancers.

## Results

### Quercetin suppressed COX-2 expression and PGE2 production

**Figure 1:**
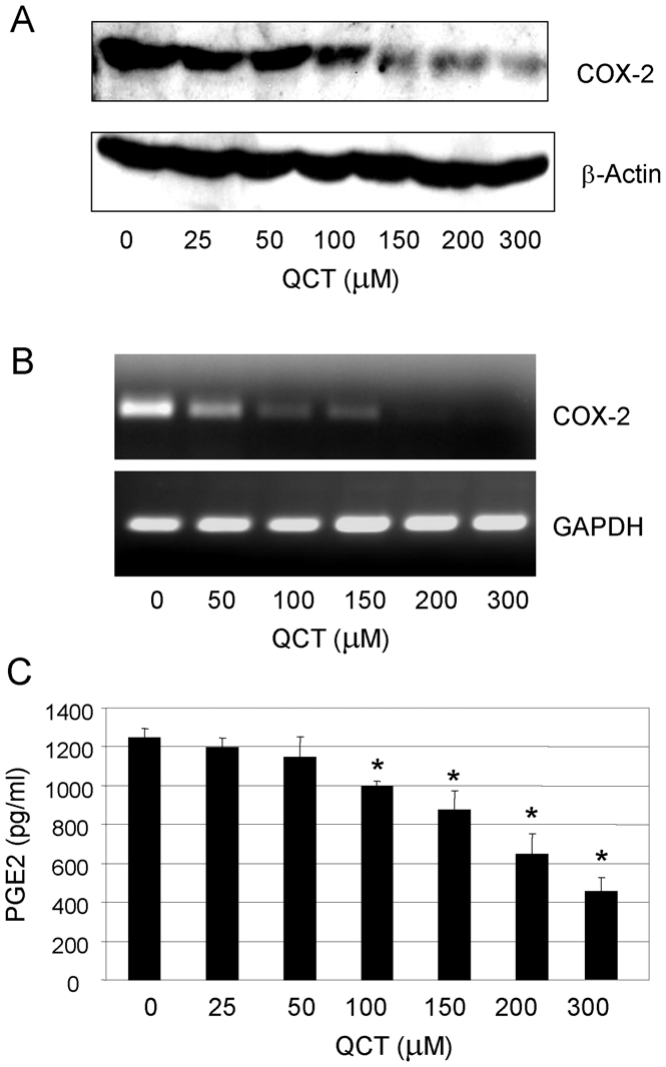
Quercetin suppressed COX-2 expression and PGE2 production. Human MDA-MB-231 cells were treated with quercetin for 48 h. The COX-2 proteins (**A**) and mRNA (**B**) were analyzed by Western blotting and RT-PCR, respectively, and PGE2 in medium of MDA-MB-231 cells was tested by ELISA (**C**). β-Actin and GAPDH were used as controls for sample loading. This is a representative of three blots. Each bar denotes mean ± SD of three experiments. *, *P*<0.05, significant differences between treatment groups and control groups.

Abnormal expression of COX-2 is an important mediator in tumor promotion. To determine whether quercetin regulated COX-2 expression in breast cancer cells, we evaluated the effect of quercetin on COX-2 protein and mRNA levels by Western blot and RT-PCR, respectively. We detected high expression of COX-2 protein (Figure 1A) and mRNA (Figure 1B) in breast cancer MDA-MB-2321 cells. Treatment of quercetin inhibited COX-2 protein expression in a concentration-dependent manner (Figure 1A). Similarly, quercetin also suppressed COX-2 mRNA levels to a similar extent in MDA-MB-231 cells (Figure 1B).

PGE2 is a downstream product of COX-2, and it is synthesized via the cyclooxygenase and prostaglandin synthase pathways. We also evaluated the effect of quercetin on PGE2 production. In accordance with inhibition of COX-2 expression, quercetin reduced PGE_2_ production in a comparable dose-dependent manner in MDA-MB-231 cells (Figure 1C).

### Quercetin inhibited COX-2 promoter activation

**Figure 2:**
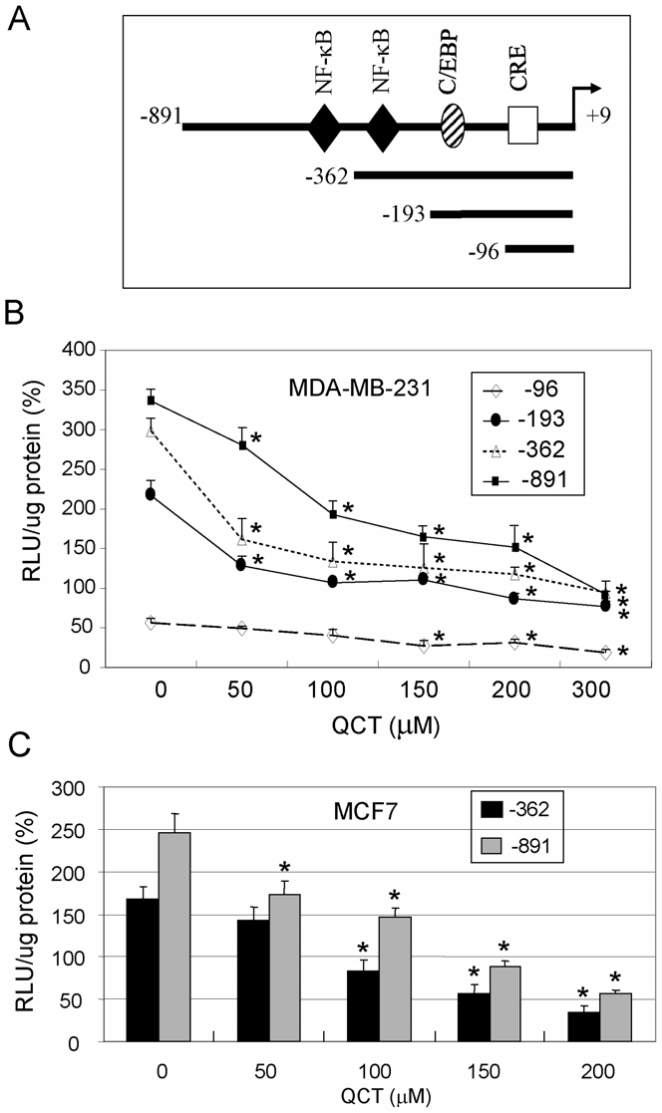
Quercetin inhibited COX-2 promoter activity. MDA-MB-231 and MCF7 cells were transfected with a luciferase expression vector containing a 900-bp wild-type COX-2 promoter or its 5-deletion mutants (**A**), and then treated with quercetin for 24 h. Luciferase activity was measured using a luminometer and the results were expressed as relative light unit (RLU) (**B**, **C**). Numbers shown for each promoter construct correspond to the COX-2 promoter sequence. Each bar denotes mean ± SD of three experiments. *, *P*<0.05, significant differences between treatment groups and control groups.

To determine whether quercetin also suppressed COX-2 transcription activation in breast cancer cells, we next determined the effects of quercetin on COX-2 promoter activity. We transfected MDA-MB-231 and MCF7 cells with a luciferase expression vector containing a 900 bp (−891/+9) COX-2 5-flanking fragment or its different deletion mutants (Figure 2A). The results showed that the promoter activity was comparably inhibited by quercetin in MDA-MB-231 cells (Figure 2B), parallel to that of COX-2 protein and mRNA suppression (Figure 1A, 1B). Treatment with quercetin dose-dependently suppressed COX-2 promoter activity in all MDA-MB-231 cells transfected with different vectors containing a 900-bp COX-2 promoter region or its deletion mutants except for the shortest mutant (−96/+9) (Figure 1B). Similarly, quercetin also significantly inhibited COX-2 promoter activity in a dose-dependent manner in MCF7 cells transfected with the vectors containing a 900-bp COX-2 promoter region (−891/+9) or its deletion mutant (−361/+9) (Figure 2C).

### Quercetin inhibited angiogenesis

**Figure 3:**
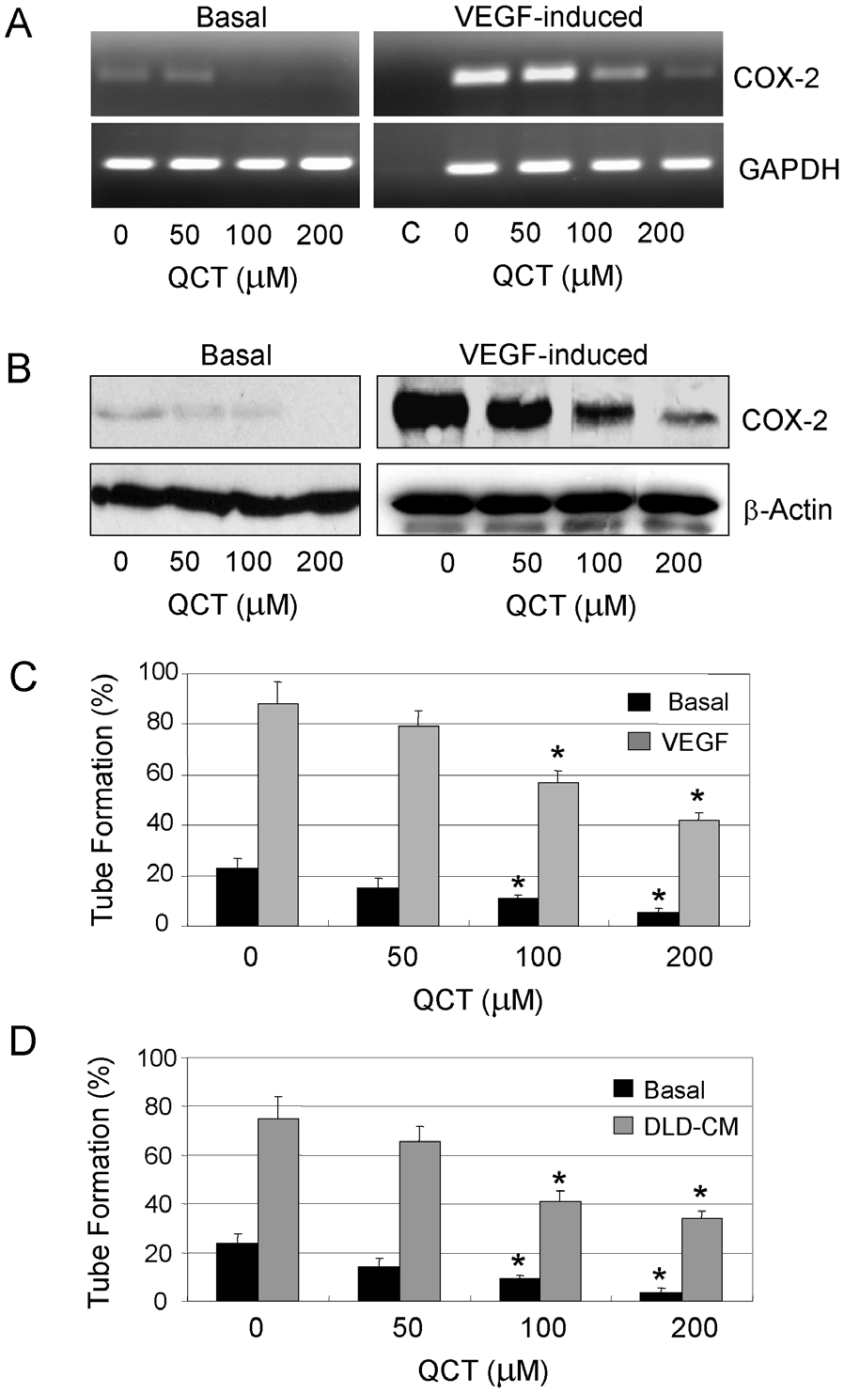
Quercetin inhibited endothelial tube formation. The HUVEC cells were plated in six-well plates pre-coated with growth factor-reduced Matrigel and treated with quercetin together with VEGF165 (10 ng/ml) or DLD-1 cancer cell medium and incubated at 37°C for 4 h and 24 h. COX-2 mRNA (**A**) and protein (**B**) were analyzed by RT-PCR and Western blotting, respectively. HUVEC tube formation was counted by phase-contrast photographs. Mean values of tube formation on growth factor-rich Matrigel induced by VEGF165 (**C**) and DLD-1 conditioned medium (**D**) were calculated from three separate experiments. The data are presented as mean ± SD of three separate experiments. *, *P*<0.05, significant differences between treatment groups and control groups. C, PCR control; Basal, medium-treated cells as negative control.

COX-2 expression and PGE2 production have been shown to induce angiogenesis, cell proliferation and invasion. We next determined the effects of quercetin on COX-2-induced angiogenesis in human endothelial cell line HUVECs by analyzing endothelial tube formation. VEGF_165_ induced COX-2 expression at mRNA (Figure 3A) and protein (Figure 3B) levels in HUVECs. Tube formation on growth factor-reduced Matrigel was also enhanced by addition of exogenous VEGF_165_ (Fig. 3C). Quercetin suppressed expression of COX-2 mRNA (Figure 3A) and protein (Figure 3B) in HUVECs induced by VEGF165. Furthermore, quercetin significantly inhibited VEGF_165_-induced tube formation in a dose-dependent manner (Figure 3C).

DLD-1 colon cancer cells have been reported to produce angiogenic factors and co-culture of these cells with endothelial cells stimulates tube formation. We next examined whether the conditioned medium (CM) from the colon cancer cells had an effect on tube formation and whether quercetin inhibited the tube formation. We washed DLD-1 cells and incubated them in serum-free medium for 24 h. The conditioned medium (CM) was collected and added to HUVECs seeded on growth factor-reduced Matrigel. As shown in Figure 3D, the DLD-1 conditioned medium (CM) induced tube formation by comparison with the basal control, and pretreatment with quercetin significantly suppressed endothelial tube formation in a concentration-dependent manner.

### Quercetin suppressed CREB-2, c-Fos, C/EBPβ, NF-κB and p300 binding to COX-2 promoter probe

**Figure 4:**
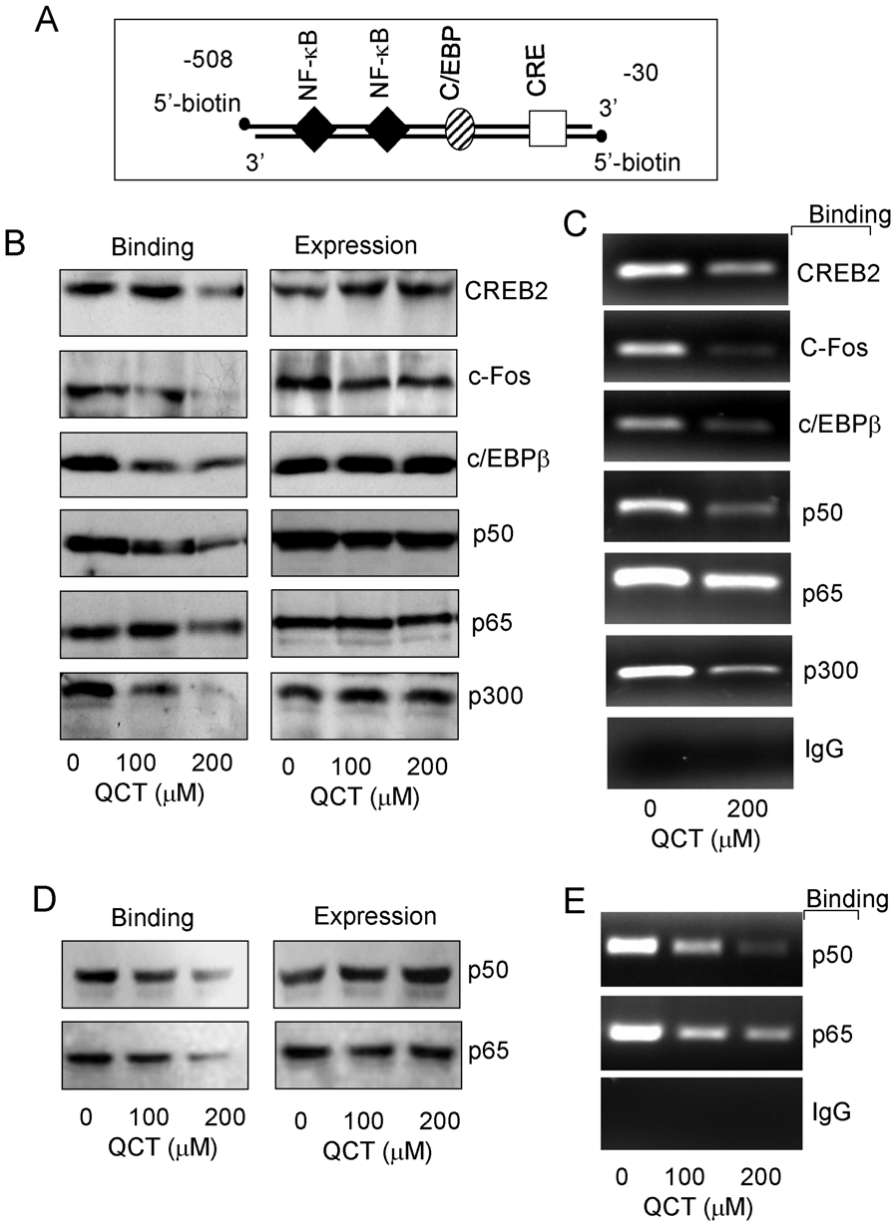
Quercetin inhibited binding of multiple transactivators and p300 to COX-2 promoter. Human MDA-MB-231 and MCF7 cells were treated with quercetin for 48 h. The binding of transactivators and p300 to COX-2 promoter was analyzed by streptavidin-agrose pulldown assay or ChIP assay. A biotin-labeled COX-2 promoter probe (**A**) which harbors all the indicated transactivators was used. Nuclear proteins in MDA-MB-231 (**B**) and MCF7 (**D**) cells were incubated with the COX-2 probe and streptavidin-agrose beads. After centrifugation, proteins in the complex were detected by immunoblots (**B**, **D**). Chromatin in MDA-MB-231 (**C**) and MCF7 (**E**) cells was immunoprecipitated with antibodies to the indicated transactivators and the COX-2 promoter region in the precipitated chromatin was amplified by PCR. The non-immuno IgG was used as negative control. The figures are representative of three experiments. IgG, non-immuno IgG.

The transcription activation of genes is regulated by the binding activities of transactivators and coactivators on gene promoter structure. Previous study has shown that quercetin suppressed expression of the proinflammatory genes IP-10 and MIP-2 by inhibiting TNF-induced phospho-RelA (NF-κB p65) and cofactor CBP/p300 recruitment to the IP-10 and MIP-2 promoters in the murine small intestinal epithelial cells [6]. To determine whether the quercetin-induced suppression of COX-2 expression is mediated by inhibition of the binding of the functionally important transactivators to COX-2 promoter in human breast cancer cells, we tested the effect of quercetin on the binding activities of CREB-2, C-Fos, C/EBPβ, NF-κB p65, and NF-κB p50 to the COX-2 promoter in MDA-MB-231 cells. We used a biotin-labeled 479-bp COX-2 promoter region corresponding to the 5- flanking sequence of human COX-2 gene from −30 to −508 as the probe to assess the binding of transactivators to COX-2 promoter by a streptavidin-agarose pulldown assay (Figure 4A). Nuclear extracts prepared from the quercetin-treated cells were incubated with a biotin-labeled COX-2 promoter probe and streptavidin-conjugated agarose beads. The biotin-streptavidin complex was pulled down and transactivators in the pulldown complex were analyzed by Western blotting. We chose to analyze the transactivators CREB-2, C-Fos, C/EBPβ and NF-κB as their binding to COX-2 promoter has been shown to be regulated. The results showed that quercetin dramatically inhibited the binding of muliple transactivators (CREB-2, c-Fos, C/EBPβ, and NF-κB p50, NF-κB p65) to the COX-2 promoter probe in human breast cancer MDA-MB-231 cells (Figure 4B). By contrast, treatment of quercetin did not change the expression levels of all transactivators (Figure 4B).

P300 is a transcription co-activator that integrates the transcriptional signal by interacting with promoter-bound transactivators such as CREB, C/EBPβ, C-Jun and NF-κB (34). Since quercetin inhibited binding of transactivators to COX-2 promoter, we suspected a consequent reduction in the level of p300 in the DNA-transactivator complex. The streptavidin-agarose bead pulldown assay showed that quercetin inhibited p300 recruitment to the transactivators-COX-2 promoter complex in MDA-MB-231 cells (Figure 4B). Quercetin did not change p300 protein expression levels (Figure 4B). These results suggest that COX-2 suppression is mediated by inhibiting transactivator binding and p300 recruitment to COX-2 promoter in MDA-MB-231 cells.

We also detected the effect of quercetin on binding activities of two key transactivators NF-κB p50 and NF-κB p65 in MCF7 breast cancer cells. Consistent with the data from MDA-MB-231 cells, treatment of MCF7 cells with quercetin also effectively inhibited p50 and p65 binding to COX-2 promoter probe, but did not change the total protein levels (Figure 4D).

### Quercetin inhibited CREB-2, c-Fos, C/EBPβ, NF-κB and p300 recruitment to chromatin COX-2 promoter region

To confirm the inhibitory effect of quercetin on transactivator binding and p300 recruitment *in vivo*, we performed chromatin immunoprecipitation (ChIP) using antibodies directed at CREB-2, c-Fos, C/EBPβ, NF-κB p65, NF-κB p50, and p300 to precipitate chromatin. The cells were treated with quercetin for 48 h and the COX-2 promoter region in the precipitated chromatin was amplified by PCR. As shown in Figure 4C, quercetin significantly inhibited binding of CREB-2, c-Fos, C/EBPβ, NF-κB p50, NF-κB p65, and p300 to chromatin COX-2 promoter in MDA-MB-231 cells. Similarly, quercetin also effectively blocked the binding of transactivators p50 and p65 to chromatin COX-2 promoter in MCF7 cells (Figure 4E). These results are in agreement with those obtained from the *in vitro* DNA-binding assays (Figure 4B and 4D).

### Quercetin inhibited p300 HAT activity

**Figure 5:**
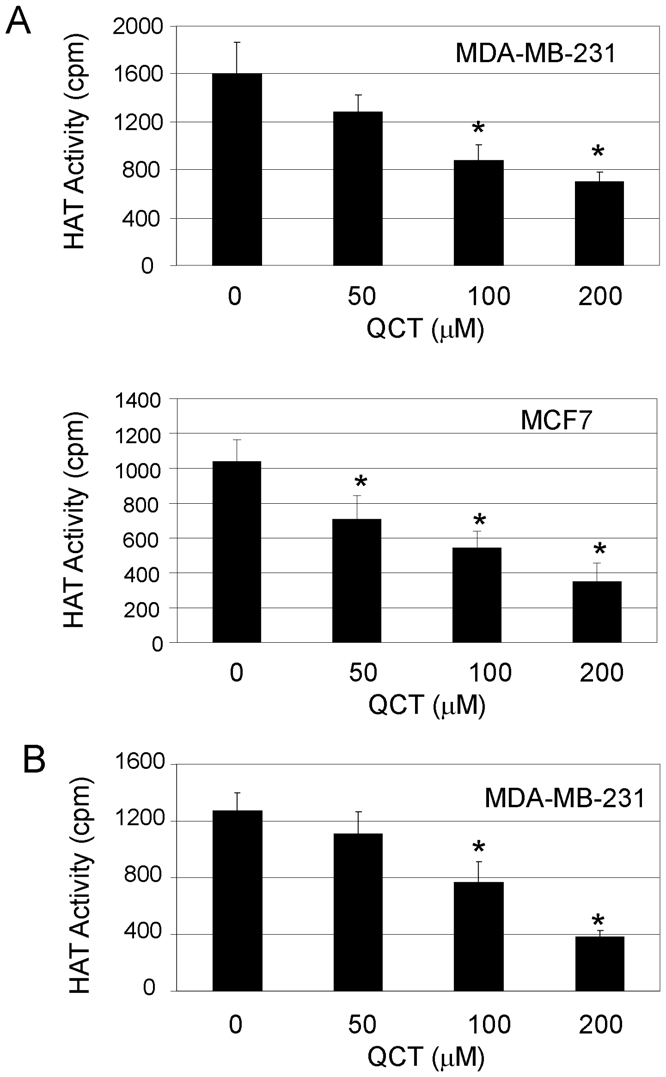
Quercetin suppressed p300 HAT activity. (**A**), Human MDA-MB-231 and MCF7 cells were transfected with FLAG-p300 for 24 h and then treated with quercetin for 24 h. The p300 protein was isolated from nuclear extracts and HAT activity was measured. (**B**), MDA-MB-231 cells were transfected with FLAG-p300. The p300 protein was isolated from nuclear extracts and then treated with quercetin *in vitro* for 30 min. HAT activity was measured. Each bar represents mean ±SD of three experiments. *, *P*<0.05, significant differences between treatment groups and control groups.

Quercetin has been shown to inhibit total HAT activity in murine small intestinal epithelial cells [6]. As quercetin exerts a global effect on transactivator binding and p300 recruitment to COX-2 promoter, we determined whether p300 HAT might be a target of transcriptional control by quercetin in human breast cancer cells and evaluated the effect of quercetin on p300 HAT activity in the p300-overexpressed breast cancer cells. We transfected MDA-MB-231 and MCF7 cells with a FLAG-p300 vector for 24 h, and then treated the p300-transfected cells with quercetin for 24 h. Nuclear extracts were immunoprecipitated with a FLAG antibody, and the overexpressed FLAG-p300 in the precipitates was eluted using FLAG peptides. HAT activity of the purified p300 was determined. Our results showed that quercetin significantly inhibited p300 HAT activity *in vivo* in a concentration-dependent manner in human breast cancer in human breast cell lines (Figure 5A).

We also determined whether quercetin directly inhibited p300 HAT activity *in vitro*. The MDA-MB-231 cells were transfected with FLAG-p300 vectors for 48 h and FLAG-p300 proteins were isolated. The purified p300 proteins were treated with quercetin and HAT activity of the purified p300 was analyzed. Quercetin also significantly inhibited p300 HAT activity *in vitro* in human breast cell lines (Figure 5B), which is comparable to its inhibition of p300 HAT *in vivo* (Figure 5A).

### Quercetin attenuated p300-mediated acetylation of NF-κB

**Figure 6:**
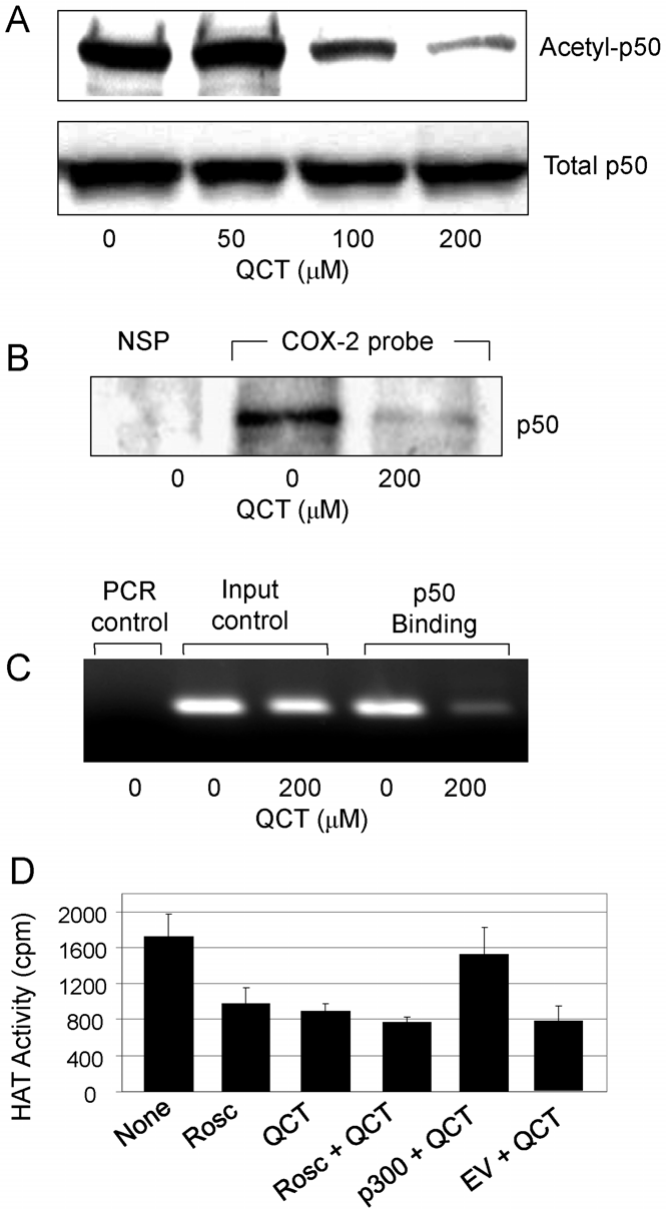
Quercetin inhibited NF-κB acetylation. (**A–C**), The MDA-MB-231 cells were transfected with FLAG-p300 for 24 h and then treated with quercetin for 24 h. Nuclear extracts were prepared and p50 was immunoprecipitated with a p50 antibody. Acetylated p50 (Actyl-p50) (**A**) was analyzed by Western blots using an acetyl-lysine antibody. The binding of p50 to a biotinylated COX-2 promoter probe (**B**) and chromatin structure (**C**) were detected by streptavidin-agarose pulldown and ChIP assay, respectively. (**D**), MDA-MB-231 cells were treated with roscovitine (20 µM) or quercetin (100 µM) for 24 h, then transfected with p300-expressing vector. After 24 h, the HAT activity was measured. The empty vector was used as a transfection control. The figures are representatives of three experiments. Each bar represents mean ±SD of three experiments. *, *P*<0.05, significant differences between treatment groups and control groups. NSP, non-specific probe; EV, empty vector.

Previous study demonstrated that quercetin inhibited TNF-induced acetylation/phosphorylation of histone (H3) in murine small intestinal epithelial cells [6]. The NF-κB p50 has been shown to be acetylated by p300 HAT. To further confirm the quercetin-mediated suppression of p300 HAT in human breast cancer cells, we evaluated the effect of quercetin on NF-κB acetylation in p300-tranfected MDA-MB-231 cells. The results showed that quercetin significantly inhibited the p300 HAT-mediated acetylation of NF-κB p50, resulting in a marked reduction in acetyl-p50 protein levels. By contrast, quercetin did not change the levels of total p50 proteins in p300-tranfected MDA-MB-231 cells (Figure 6A). Streptavidin-agarose pulldown and ChIP assay also showed that quercetin dramatically suppressed binding of p50 to the biotinylated COX-2 promoter probe (Figure 6B) and the chromatin COX-2 promoter region (Figure 6C) in p300-tranfected MDA-MB-231 cells.

To confirm the inhibition of p300 signaling by quercetin, we treated MDA-MB-231 cells with the p300 inhibitor roscovitine (20 µM) in the presence or absence of quercetin, and evaluated their effects on p300 HAT inhibition. As shown in Figure 6D, roscovitine was as effective as quercetin at inhibiting p300 HAT activity. Addition of quercetin to the roscovitine-treated cells did not change the inhibition of p300 HAT mediated by roscovitin (Figure 6D). However, transfection of p300 expressing vector in the quercetin-pretreated cells significantly reversed inhibition of the endogenous HAT activity mediated by quercetin (Figure 6D). Taken together, these results indicate that p300 HAT is an important target of transcriptional control by quercetin. Quercetin directly inhibits p300 HAT, thereby attenuating the acetylation and binding of transactivators and suppressing the expression of COX-2.

## Discussion

In this study, we evaluated the response of human breast cancer cells to quercetin treatment. Our results showed that quercetin significantly suppressed COX-2 expression and PGE2 production, as well as COX-2 promoter activation. We also found that quercetin significantly inhibited angiogenesis by suppressing endothelial tube formation in human HUVEC cells. Furthermore, mechanic study showed that suppression of COX-2 expression by quercetin is mediated mainly by inhibiting p300 HAT activity, thereby attenuating the acetylation of transactivators and their binding to COX-2 promoter. Our results indicate that quercetin may target the p300 signaling to inhibit COX-2 expression in human breast cancer cells.

Quercetin has been shown to inhibit TNF-induced expression of the proinflammatory genes IP-10 and MIP-2 by modulating TNF-induced NF-kB signaling in murine intestinal epithelial cells (IECs) [6]. Quercetin reduces total HAT activity, blocks TNF-induced acetylation and/or phosphorylation of histone 3 (H3), and inhibits the recruitment of the NF-*κ*B cofactor CBP/p300 to the IP-10 and MIP-2 gene promoters. These results suggest that quercetin may target the TNF-induced transcriptional regulation at the chromatin. However, whether quercetin may specifically target p300 signaling pathway to regulate expression of the inflammation and cancer-related genes remains unknown.

COX-2 is a specific proinflammatory gene. Excessive COX-2 expression plays a key role in inflammatory disorders and cancers. In this study, we revealed the molecular mechanisms by which quercetin inhibited COX-2 expression and COX-2-mediated angiogenesis in human breast cancer cell lines. Unlike the quercetin-mediated regulation of IP-10 and MIP-2 gene expression in murine intestinal epithelial cells, our results showed that quercetin suppressed COX-2 expression in human breast cancer cells by specifically targeting the p300 signaling pathway. We found that quercetin specifically inhibited p300 HAT activity *in vitro* and *in vivo*, blocked p300 HAT-mediated acetylation of NF-*κ*B p50, and inhibited the binding of p300 to COX-2 promoter probe *in vitro* and to chromatin COX-2 promoter region *in vivo* in human breast cells. Interestingly, and most important for understanding the mechanism involved, quercetin dramatically inhibited the recruitment of multiple transactivators such as CREB-2, c-Fos, C/EBPβ, NF-κB to COX-2 promoter, suggesting that quercetin may target the p300 signaling and modulate the binding activities of the functionally important transactivators at the promoter to regulate COX-2 transcriptional activation. To our knowledge, this is an important finding that demonstrated suppression of p300 signaling by quercetin in human breast cancer cells.

COX-2 expression and PGE2 production have been shown to upregulate the EGFR, PI3k and Erk1/2 signaling, thereby inducing angiogenesis, cell proliferation and invasion. COX-2 inhibition may result in cell growth suppression and apoptosis induction. The AMP kinase/cyclooxygenase-2 signaling pathway has been shown to regulate proliferation and apoptosis of cancer cells treated with quercetin and is important in quercetin-mediated cancer control. Quercetin activates AMP-activated protein kinase (AMPK) and inhibits cyclooxygenase (COX-2) expression in breast and colon cancer cell lines [13].

COX-2 promoter activation is mediated by binding of multiple transactivators to enhancer elements of COX-2 promoter. Results from previous studies show that the CRE site, C/EBP site, and two κB sites are essential for COX-2 promoter activity [34], [35]. It has been shown that CREB-2 and AP-1 (c-Jun and c-Fos) bind to the CRE and its surrounding region, C/EBPβ binds to the C/EBP site, and NF-κB binds to both κB sites. We designed a COX-2 promoter region that includes all four binding sites as a probe to study the effects of quercetin on COX-2 transcription activation. We demonstrated the inhibitory effects of quercetin on the binding of transactivators CREB-2, c-Fos, C/EBPβ and NF-κB to COX-2 promoter *in vitro* by streptavidin-agarose pulldown. We also showed by *in vivo* ChIP assay that quercetin suppressed binding of transactivators to chromatin COX-2 promoter structure. The transactivators recruit p300 coactivator to COX-2 promoter where the coactivators interact with the transcriptional machinery and thereby integrate the signal for promoter activation. We provide evidence in this study that quercetin also inhibited p300 recruitment to the COX-2 promoter. Taken together, our data suggest that quercetin inhibits COX-2 expression in breast cancer cells by suppressing transactivator DNA binding activities, thereby compromising p300 recruitment and resulting in suppression of COX-2 promoter activity. The mechanisms by which quercetin blocks transactivator binding remain to be unclear. Previous study showed quercetin inhibited TNF-induced NF-κB transcription factor recruitment to proinflammatory gene promoters in murine intestinal epithelial cells [6]. As the DNA binding activities of transactivators such as AP-1, C/EBPβ and NF-κB are regulated by phosphorylation via multiple kinase signaling pathways [41], it is possible that quercetin may suppress transactivator binding by inhibiting a common kinase pathway.

P300 is expressed in abundance in cancer cells and p300 overexpression augments COX-2 transcriptional activation induced by diverse pro-inflammatory mediators [35]. It serves as a transcription coactivator to bridge the promoter-bound transactivators with transcriptional factors in the transcription machinery. P300 HAT acetylates histones thereby opening the chromatin structure and increasing access of enhancer elements to transactivators. P300 HAT is also capable of acetylating transactivators such as NF-κB, thereby enhancing transactivator binding [34], [35]. P300 HAT has been shown to be essential for COX-2 promoter activation. The p300 HAT deletion or HAT inhibitors abrogate the stimulatory effect of diverse pro-inflammatory mediators on COX-2 expression [34], [35]. Importantly, p300 HAT exerts a global effect on COX-2 promoter chromatin structure to enhance binding of transactivators. Consistent with the previous reports, our present study have also showed that p300 HAT plays a crucial role in regulating COX-2 expression. We found that quercetin suppresses COX-2 expression mainly by inhibiting p300-mediated acetylation of transactivators in human breast cancer cells. However, the role of quercetin in regulating histone acetylation on COX-2 promoter remains unclear and further studies are needed.

That quercetin inhibits p300 HAT activity suggests that quercetin targets at p300 HAT, thereby blocking the access of transactivators and p300 to the COX-2 promoter region. Quercetin may inhibit the catalytic center of p300 HAT. It also may block p300 HAT activation by post-translational modification. It has been shown that p300 HAT is regulated by p300 phosphorylation or acetylation [42], [43]. It is possible that quercetin suppresses p300 phosphorylation, alters p300 HAT conformation and catalytic activity, and inhibits the post translational modification of p300 HAT, thereby blocking HAT activity in breast cancer cells. Previous study also showed that histone acetylation by p300 is involved in CREB-mediated transcription on chromatin [43]. Further studies are needed to elucidate the mechanism by which quercetin inhibits p300 HAT activity.

The transcriptional control by quercetin in breast cancer cells is likely not limited to COX-2 gene but also other genes, especially the pro-inflammatory, mitogenic and oxygenases whose promoter activities are regulated by p300 HAT and whose overexpression cause oxidative DNA damage and the consequent mutation and cellular changes. Our results should light on an important transcriptional control program mediated by quercetin in breast cancer cells. Our findings provide new insights into understanding the molecular mechanisms of quercetin-mediated tumor suppression and suggest that flavonoids such as quercetin may be effective agents for the treatment of COX-2-medaited human diseases such as cancers.

## Materials and Methods

### Cell lines and cell culture

Human breast cancer cells MDA-MB-231, MCF7 and colon cancer cells DLD-1, and primary human umbilical vein endothelial cells (HUVECs) were obtained from American Type Culture Collection (ATCC, Manassas, VA). The MDA-MD-231 and DLD-1 cells were cultured in RPMI1640 medium (Invitrogen, Carlsbad, CA), supplemented with 10% fetal bovine serum (FBS) (HyClone, Logan, UT), 5% glutamine, 100 U/ml penicillin and 100 µg/ml streptomycin (Invitrogen, Carlsbad, CA). The primary HUVECs were maintained in medium M199 (Life Technologies, Gaithersburg, MD) containing 4 mM L-glutamine, 90 µg/ml heparin, 1 mM sodium pyruvate, 30 µg/ml endothelial cell growth stimulant (Biomedical Products, Bedford, MA), and 20% FBS (HyClone, Logan, UT). Only cells at passage 2 to 4 were used in our experiments. All cell lines were grown at 37°C in an atmosphere of 5% CO_2_. In all experiments, 80–90% of confluent cells were washed and incubated in serum-free medium for 24 h prior to treatment with quercetin (Sigma, St. Louis, MO) or PD098059 (Cell Signaling Technology, Beverly, MA) for 48 h.

### Western blot analysis

Cell lysate proteins were separated by electrophoresis in a 4–15% sodium dodecyl suplhaste-polyacrylamide gradient minigel (SDS-PAGE) (Bio-Rad, Hercules, CA) and electrophoretically transferred to a nitrocellulose membrane (Amersham Pharmacia Biotech, Piscataway, NJ). Western blots were probed with antibodies against COX-2, CREB2, C-Jun, C/EBPβ, p50, p65, p300 (Santa Cruz Biotechnology, Santa Cruz, CA). The protein bands were detected by enhanced chemiluminescence (Amersham Pharmacia Biotech, Piscataway, NJ).

### RT-PCR

Total RNA was extracted with Tri-Zol reagent (Life Technologies, Glasgow, UK) according to the manufacturer's instructions. cDNA was synthesized and used for amplification of COX-2 gene. The amplified products were visualized on 1% agarose gels.

### PGE_2_ assay

Amounts of PGE*_2_* in the conditioned media collected from the quercetin-treated cells were determined by an enzyme-linked immunosorbent assay according to the instructions of the manufacturer (Amersham Pharmacia Biotech, Piscataway, NJ).

### Analysis of promoter activity

A promoter region of human COX-2 gene (−891 to +9) and its fragment deletions (Figure 2D) were constructed into a luciferase reporter vector pGL3. The COX-2 promoter activity was determined by transient expression of the luciferase vectors in MDA-MB-231 cells. In brief, 4 µg of the promoter vector was mixed with 10 µl of Lipofetamine 2000 (Invitrogen, Carlsbad, CA) and the mixture was slowly added to cells in a 6-well plate (200,000 cells/well) and incubated for 24 h. The cells were then washed, incubated in serum free medium for 24 h and treated with quercetin for 24 h. Cells were lysed and luciferase activity was measured using an assay kit (Promega, Madison, WI).

### In vitro angiogenesis assay

HUVECs were used for angiogenesis assay according to a previously described method [44]. HUVECs were grown in 100-mm dishes until they were 80% confluent. The cells were trypsinized, counted, and resuspended in serum-free medium at a concentration of 4×10^4^ cells/ml. Six-well plates were evenly coated with growth factor-reduced Matrigel (Becton Dickinson, Milan, Italy), which was allowed to solidify at 37°C for 30 min before endothelial cells were plated. Quercetin or vehicle control was added together with angiogenic factors or cancer cell medium to each well and incubated at 37°C for 4 h and 24 h. Tube formation was quantified by counting the number of connected cells in five randomly selected fields by using 200× magnification and dividing that number by the total number of cells in the same field.

### DNA-protein binding by streptavidin-agarose pulldown assay

Transactivator and p300 binding to a COX-2 core promoter probes were determined by a streptavidin-agarose pulldown assay as previously described [45], [46]. This assay allows for simultaneous quantitative determination of transactivators and coactivators that complex with the DNA probes. A 478-bp biotin-labeled double-stranded probe corresponding to COX-2 promoter sequence (−30 to −508) was synthesized. The sequence probe contains all the sites required for COX-2 promoter activation. The binding assay was performed by mixing 400 µg of nuclear extract proteins, 4 µg of the biotinylated DNA probe and 40 µl of 4% streptavidin-conjugated agarose beads at room temperature for 1 h in a rotating shaker. Beads were pelleted by centrifugation to pull down the DNA-protein complex. After washing, proteins in the complex were analyzed by immunoblots using rabbit polyclonal antibodies (1 µg/ml each) specific for the indicated transcription factors. A nonrelevant biotinylated sequence 5′-AGAGTGGTCACTACCCCCTCTG-3′ was included as a non-specific control probe (NSP). The mixture was incubated at room temperature for 1 h with shaking, and centrifuged to pull down the DNA-protein complex. DNA-bound transactivators or p300 were dissociated and analyzed by Western blotting. A non-immune rabbit IgG (1 µg/ml) was also used as negative controls.

### Chromatin Immunoprecipitation (ChIP)

The ChIP assay was performed as previously described with minor modifications (34). In brief, ∼80% confluent cells were serum-starved for 24 h and treated with or without quercetin at 37°C for 48 h. 1% formaldehyde was added to the culture medium, and after incubation for 20 min at 37°C, the cells were washed twice in phosphate-buffered saline, scraped, and lysed in lysis buffer (1% SDS, 10 mM Tris-HCl, pH 8.0,with 1 mM phenylmethylsulfonyl fluoride, pepstatin A, and aprotinin) for 10 min at 4°C. The lysates were sonicated five times for 10 s each time, and the debris was removed by centrifugation. One-third of the lysate was used as DNA input control. The remaining two-thirds of the lysate were diluted 10-fold with a dilution buffer (0.01% SDS, 1% Triton X-100, 1 mM EDTA, 10 mM Tris-HCl, pH 8.0, and 150 mM NaCl) followed by incubation with antibodies against specific transactivators or a nonimmune rabbit IgG control overnight at 4°C. Immunoprecipitated complexes were collected by using protein A/G plus agarose beads. The precipitates were extensively washed and incubated in an elution buffer (1% SDS and 0.1 M NaHCO_3_) at room temperature for 20 min. Cross-linking of protein-DNA complexes was reversed at 65°C for 5 h, followed by treatment with 100 µg/ml proteinase K for 3 h at 50°C. DNA was extracted three times with phenol/chloroform and precipitated with ethanol. The pellets were resuspended in TE buffer and subjected to PCR amplification using specific COX-2 promoter primers (5′ primer, ^–709^ CTGTTGAAAGCAACTTAGCT ^–690^, and 3′ primer ^–32^ AGACTGAAAACCAAGCCCAT ^–51^). The resulting product of 678 bp for COX-2 in length was separated by agarose gel electrophoresis.

### Analysis of HAT activity

Cells were transfected with a FLAG-p300 vector for 24 h and treated with quercetin for 24 h. The nuclear extracts prepared from the treated cells were immunoprecipitated with an anti-FLAG M2 affinity gel (Sigma, St. Louis, MO). FLAG-p300 was eluted with FLAG peptides and the HAT activity of the purified FLAG-p300 was analyzed. FLAG-p300 were incubated for 30 min at 30°C in 100 µl HAT buffer (100 mM Tris HCl pH 8, 20% glycerol, 2 mM DTT, 2 mM PMSF, 0.2 mM EDTA, 0.1 M NaCl and 0.02 M butyric acid) containing 100 µg histone (Sigma, St. Louis, MO) and 35 pmol ^3^H-acetyl Coenzyme A (Amersham, Piscataway, NJ). The samples were spotted on P81 phosphocellulose squares (Upstate, Lake Placid, NY) and [^3^H] acetyl histone was measured in a scintillation counter.

### Acetylation analysis

Acetylation of p50 was determined as described previously (41). Briefly, p50 in nuclear extracts was immunoprecipitated with a specific antibody of p50 and the immunoprecipitate was pulled down with protein A/G agarose beads (Santa Cruz Biotechnology, Santa Cruz, CA). After extensive washing, p50 proteins were separated in a 4–20% SDS-PAGE system and acetylated p50 was detected with a monoclonal antibody against acetyl-lysine (1∶1000 dilution) (Cell Signaling Tech., Beverly, MA).

### Statistical analysis

Analysis of variance and Student's *t* test were used to compare the values of the test and control samples. *P<0.05* was considered to a statistically significant difference. StatView 5.0 (Abacus Concepts, Inc., Berkeley, CA) and SAS software were used for all statistical analyses. The significance was evaluated by the paired *t* test. All the experiments were done three times, and mean values and standard deviation were calculated.

## References

[1] Linsalata M, Orlando A, Messa C, Refolo MG, Russo F (2010). Quercetin inhibits human DLD-1 colon cancer cell growth and polyamine biosynthesis.. Anticancer Res.

[2] Russo M, Spagnuolo C, Volpe S, Mupo A, Tedesco I (2010). Quercetin induced apoptosis in association with death receptors and fludarabine in cells isolated from chronic lymphocytic leukaemia patients.. Br J Cancer.

[3] Thangasamy T, Sittadjody S, Mitchell GC, Mendoza EE, Radhakrishnan VM (2010). Quercetin abrogates chemoresistance in melanoma cells by modulating deltaNp73.. BMC Cancer.

[4] Nguyen TT, Tran E, Nguyen TH, Do PT, Huynh TH (2004). The role of activated MEK-ERK pathway in quercetin-induced growth inhibition and apoptosis in A549 lung cancer cells.. Carcinogenesis.

[5] Boots AW, Wilms LC, Swennen EL, Kleinjans JC, Bast A (2008). In vitro and ex vivo anti-inflammatory activity of quercetin in healthy volunteers.. Nutrition.

[6] Ruiz PA, Braune A, Holzlwimmer G, Quintanilla-Fend L, Haller D (2007). Quercetin inhibits TNF-induced NF-kappaB transcription factor recruitment to proinflammatory gene promoters in murine intestinal epithelial cells.. J Nutr.

[7] Lee YK, Park OJ (2010). Regulation of mutual inhibitory activities between AMPK and Akt with quercetin in MCF-7 breast cancer cells.. Oncol Rep.

[8] Chou CC, Yang JS, Lu HF, Ip SW, Lo C (2010). Quercetin-mediated cell cycle arrest and apoptosis involving activation of a caspase cascade through the mitochondrial pathway in human breast cancer MCF-7 cells.. Arch Pharm Res.

[9] Senthilkumar K, Elumalai P, Arunkumar R, Banudevi S, Gunadharini ND (2010). Quercetin regulates insulin like growth factor signaling and induces intrinsic and extrinsic pathway mediated apoptosis in androgen independent prostate cancer cells (PC-3).. Mol Cell Biochem.

[10] Du G, Lin H, Yang Y, Zhang S, Wu X (2010). Dietary quercetin combining intratumoral doxorubicin injection synergistically induces rejection of established breast cancer in mice.. Int Immunopharmacol.

[11] Jung YH, Heo J, Lee YJ, Kwon TK, Kim YH (2010). Quercetin enhances TRAIL-induced apoptosis in prostate cancer cells via increased protein stability of death receptor 5.. Life Sci.

[12] Du G, Lin H, Wang M, Zhang S, Wu X (2009). Quercetin greatly improved therapeutic index of doxorubicin against 4T1 breast cancer by its opposing effects on HIF-1alpha in tumor and normal cells.. Cancer Chemother Pharmacol.

[13] Lee YK, Park SY, Kim YM, Lee WS, Park OJ (2009). AMP kinase/cyclooxygenase-2 pathway regulates proliferation and apoptosis of cancer cells treated with quercetin.. Exp Mol Med.

[14] Choi EJ, Bae SM, Ahn WS (2008). Antiproliferative effects of quercetin through cell cycle arrest and apoptosis in human breast cancer MDA-MB-453 cells.. Arch Pharm Res.

[15] Chen W, Wang X, Zhuang J, Zhang L, Lin Y (2007). Induction of death receptor 5 and suppression of survivin contribute to sensitization of TRAIL-induced cytotoxicity by quercetin in non-small cell lung cancer cells.. Carcinogenesis.

[16] Vijayababu MR, Arunkumar A, Kanagaraj P, Venkataraman P, Krishnamoorthy G (2006). Quercetin downregulates matrix metalloproteinases 2 and 9 proteins expression in prostate cancer cells (PC-3).. Mol Cell Biochem.

[17] Gulati N, Laudet B, Zohrabian VM, Murali R, Jhanwar-Uniyal M (2006). The antiproliferative effect of Quercetin in cancer cells is mediated via inhibition of the PI3K-Akt/PKB pathway.. Anticancer Res.

[18] Vijayababu MR, Arunkumar A, Kanagaraj P, Arunakaran J (2006). Effects of quercetin on insulin-like growth factors (IGFs) and their binding protein-3 (IGFBP-3) secretion and induction of apoptosis in human prostate cancer cells.. J Carcinog.

[19] Yang JH, Hsia TC, Kuo HM, Chao PD, Chou CC (2006). Inhibition of lung cancer cell growth by quercetin glucuronides via G2/M arrest and induction of apoptosis.. Drug Metab Dispos.

[20] Kim WK, Bang MH, Kim ES, Kang NE, Jung KC (2005). Quercetin decreases the expression of ErbB2 and ErbB3 proteins in HT-29 human colon cancer cells.. J Nutr Biochem.

[21] Howe LR (2007). Inflammation and breast cancer. Cyclooxygenase/prostaglandin signaling and breast cancer.. Breast Cancer Res.

[22] Sinicrope FA, Gill S (2004). Role of cyclooxygenase-2 in colorectal cancer.. Cancer Metastasis Rev.

[23] Singh B, Lucci A (2002). Role of cyclooxygenase-2 in breast cancer.. J Surg Res.

[24] Castellone MD, Teramoto H, Williams BO, Druey KM, Gutkind JS (2005). Prostaglandin E2 promotes colon cancer cell growth through a Gs-axin-beta-catenin signaling axis.. Science.

[25] Stachowska E, Dolegowska B, Dziedziejko V, Rybicka M, Kaczmarczyk M (2009). Prostaglandin E2 (PGE2) and thromboxane A2 (TXA2) synthesis is regulated by conjugated linoleic acids (CLA) in human macrophages.. J Physiol Pharmacol.

[26] Onguru O, Casey MB, Kajita S, Nakamura N, Lloyd RV (2005). Cyclooxygenase-2 and thromboxane synthase in non-endocrine and endocrine tumors: a review.. Endocr Pathol.

[27] Kajita S, Ruebel KH, Casey MB, Nakamura N, Lloyd RV (2005). Role of COX-2, thromboxane A2 synthase, and prostaglandin I2 synthase in papillary thyroid carcinoma growth.. Mod Pathol.

[28] Kang CH, Chiang PH, Huang SC (2008). Correlation of COX-2 expression in stromal cells with high stage, high grade, and poor prognosis in urothelial carcinoma of upper urinary tracts.. Urology.

[29] de ME, Dar NA, de Moura Gallo CV, Hainaut P (2007). Cross-talks between cyclooxygenase-2 and tumor suppressor protein p53: Balancing life and death during inflammatory stress and carcinogenesis.. Int J Cancer.

[30] Wang D, Dubois RN (2004). Cyclooxygenase-2: a potential target in breast cancer.. Semin Oncol.

[31] de Groot DJ, de Vries EG, Groen HJ, de JS (2007). Non-steroidal anti-inflammatory drugs to potentiate chemotherapy effects: from lab to clinic.. Crit Rev Oncol Hematol.

[32] Sanborn R, Blanke CD (2005). Cyclooxygenase-2 inhibition in colorectal cancer: boom or bust?. Semin Oncol.

[33] Fu SL, Wu YL, Zhang YP, Qiao MM, Chen Y (2004). Anti-cancer effects of COX-2 inhibitors and their correlation with angiogenesis and invasion in gastric cancer.. World J Gastroenterol.

[34] Deng WG, Zhu Y, Wu KK (2004). Role of p300 and PCAF in regulating cyclooxygenase-2 promoter activation by inflammatory mediators.. Blood.

[35] Deng WG, Zhu Y, Wu KK (2003). Up-regulation of p300 binding and p50 acetylation in tumor necrosis factor-alpha-induced cyclooxygenase-2 promoter activation.. J Biol Chem.

[36] Deng WG, Tang ST, Tseng HP, Wu KK (2006). Melatonin suppresses macrophage cyclooxygenase-2 and inducible nitric oxide synthase expression by inhibiting p52 acetylation and binding.. Blood.

[37] Wu KK (2003). Control of COX-2 and iNOS gene expressions by aspirin and salicylate.. Thromb Res.

[38] Lee KM, Hwang MK, Lee DE, Lee KW, Lee HJ (2010). Protective effect of quercetin against arsenite-induced COX-2 expression by targeting PI3K in rat liver epithelial cells.. J Agric Food Chem.

[39] Turner ND, Paulhill KJ, Warren CA, Davidson LA, Chapkin RS (2009). Quercetin suppresses early colon carcinogenesis partly through inhibition of inflammatory mediators.. Acta Hortic.

[40] Chiu WT, Shen SC, Chow JM, Lin CW, Shia LT (2010). Contribution of reactive oxygen species to migration/invasion of human glioblastoma cells U87 via ERK-dependent COX-2/PGE(2) activation.. Neurobiol Dis.

[41] Persichini T, Maio N, di Patti MC, Rizzo G, Colasanti M, Musci G (2010). Interleukin-1beta induces ceruloplasmin and ferroportin-1 gene expression via MAP kinases and C/EBPbeta, AP-1, and NF-kappaB activation.. Neurosci Lett.

[42] Brouillard F, Cremisi CE (2003). Concomitant increase of histone acetyltransferase activity and degradation of p300 during retinoic acid-induced differentiation of F9 cells.. J Biol Chem.

[43] Yuan LW, Gambee JE (2001). Histone acetylation by p300 is involved in CREB-mediated transcription on chromatin.. Biochim Biophys Acta.

[44] Deng WG, Saunders MA, Gilroy DW, He XZ, Yeh H (2002). Purification and characterization of a cyclooxygenase-2 and angiogenesis suppressing factor produced by human fibroblasts.. FASEB J.

[45] Deng WG, Montero AJ, Wu KK (2007). Interferon-gamma suppresses cyclooxygenase-2 promoter activity by inhibiting C-Jun and C/EBPbeta binding.. Arterioscler Thromb Vasc Biol.

[46] Deng WG, Zhu Y, Montero AJ, Wu KK (2003). Quantitative analysis of binding of transcription factor complex to biotinylated DNA probe by a streptavidin-agarose pulldown assay.. Anal Biochem.

